# PICC insertion in the sitting position for a patient with congestive heart failure: A case report

**DOI:** 10.1097/MD.0000000000014413

**Published:** 2019-02-08

**Authors:** Shingo Mitsuda, Joho Tokumine, Rena Matsuda, Tomoko Yorozu, Takayuki Asao

**Affiliations:** a Department of Anesthesiology, Kyorin University School of Medicine, Sinkawa, Mitaka, Tokyo; b Big Data Center for Integrative Analysis, Gunma University Initiative for Advance Research, Showa, Maebashi, Gunnma, Japan.

**Keywords:** congestive heart failure, peripherally inserted central catheter, sitting position

## Abstract

**Rationale:**

A peripherally inserted central catheter (PICC) is typically inserted with the patient in the supine position. Here, we placed a PICC in a patient in the sitting position, in order to treat congestive heart failure.

**Patient concerns:**

A 65-year-old man was diagnosed with end-stage lung cancer. He had experienced septic shock and was medicated with continuous infusion of noradrenaline through a peripheral vein, in order to maintain sufficient blood pressure. However, indwelling peripheral venous catheters were difficult to place and maintain.

**Diagnosis:**

The patient experienced orthopnea due to congestive heart failure and could not assume any other position.

**Interventions:**

An anesthesiologist performed PICC placement while the patient was in the sitting position, using ultrasound guidance.

**Outcomes:**

The patient's orthopnea was slightly ameliorated, and he was able to sleep at night.

**Lessons:**

The technique of inserting a PICC in the sitting position is simple and feasible. This approach may be useful for patients in whom central venous access is needed, but the supine position cannot be achieved.

## 1 Introduction

In some cases of congestive heart failure, central venous catheters are used to continuously administer inotropes.^[1]^ Recently, a peripherally inserted central catheter (PICC) approach has been used instead of a central venous catheter approach.^[2]^

Typically, a PICC is inserted in the supine position. A PICC may not be appropriate for a patient with congestive heart failure in the acute symptomatic phase, due to orthopnea. We placed a PICC in a patient in the sitting position, in order to treat congestive heart failure. This is the first report of insertion of a PICC in a patient in the sitting position.

## 2 Consent statement

Written informed consent was obtained from the patient's family for the publication of this case report.

## 3 Case report

A 65-year-old man was diagnosed with pulmonary adenocarcinoma, then treated with chemotherapy. The cancer metastasized, and the patient was diagnosed with end-stage lung cancer. Cancer pain was treated with oral opioid therapy. Metastasis of the cancer caused obstructive jaundice and cholangitis, and the patient experienced septic shock. The attending physician performed emergent endoscopic biliary stent placement, in order to treat obstructive jaundice. The patient was medicated with continuous infusion of noradrenaline (0.2 μg/kg/min) through a peripheral vein, in order to maintain blood pressure. He underwent repeated placement of peripheral venous catheters, such that it became difficult to identify adequate peripheral veins for placement and maintenance of indwelling catheters. In the context of this treatment, the patient's congestive heart failure continued, which caused ascites and edema of the lower extremities.

The patient experienced orthopnea and could not sleep at night. The attending physician requested that the anesthesiologists secure a reliable venous line. The anesthesiologists checked the patient's groin and thighs; they excluded the femoral vein as a candidate for central venous access due to massive edema of the thighs. The anesthesiologists attempted to allow the patient to assume the supine or reverse Trendelenburg position; however, these positions caused worsening of the patient's dyspnea. The patient could solely tolerate the sitting position. The anesthesiologists then discontinued attempts to catheterize via the internal jugular and subclavian veins, because of the risk of air embolism during central venous catheterization of the patient in the sitting position.

**Figure 1:**
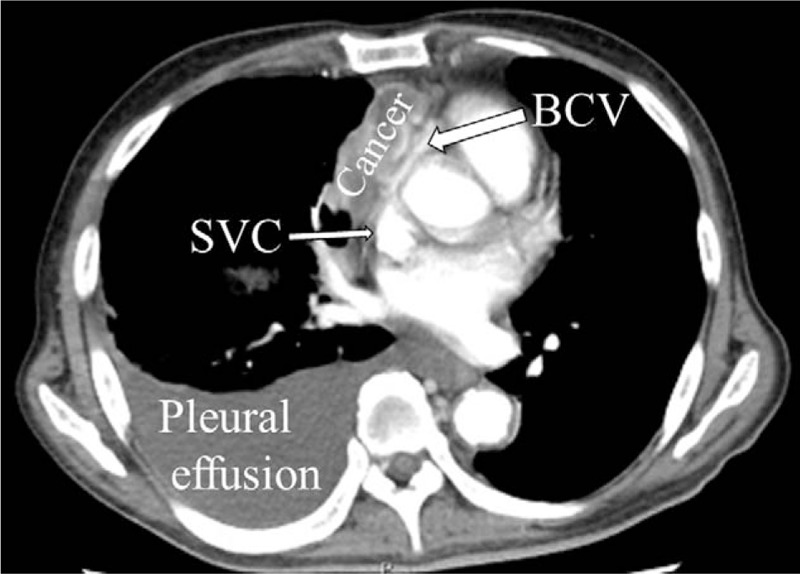
Computed tomography of the patient's chest. Lung cancer occupies the right upper mediastinum. The left brachiocephalic vein shows narrowing, and the right brachiocephalic vein cannot be identified. BCV = left brachiocephalic vein, cancer = lung adenocarcinoma, SVC = superior vena cava.

**Figure 2:**
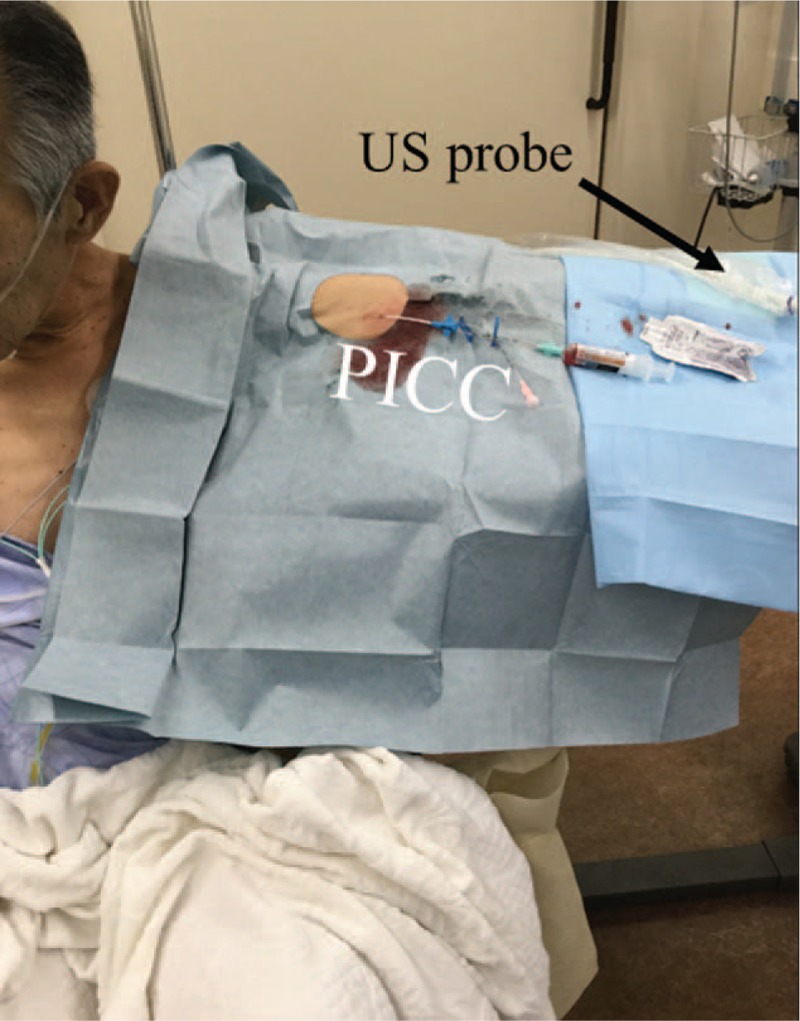
Placement of a peripherally inserted central catheter while the patient remained in the sitting position. The patient could only assume the sitting position due to orthopnea. Therefore, a peripherally inserted central catheter was inserted while the patient remained in the sitting position. PICC = peripherally inserted central catheter, US probe = ultrasound probe.

**Figure 3:**
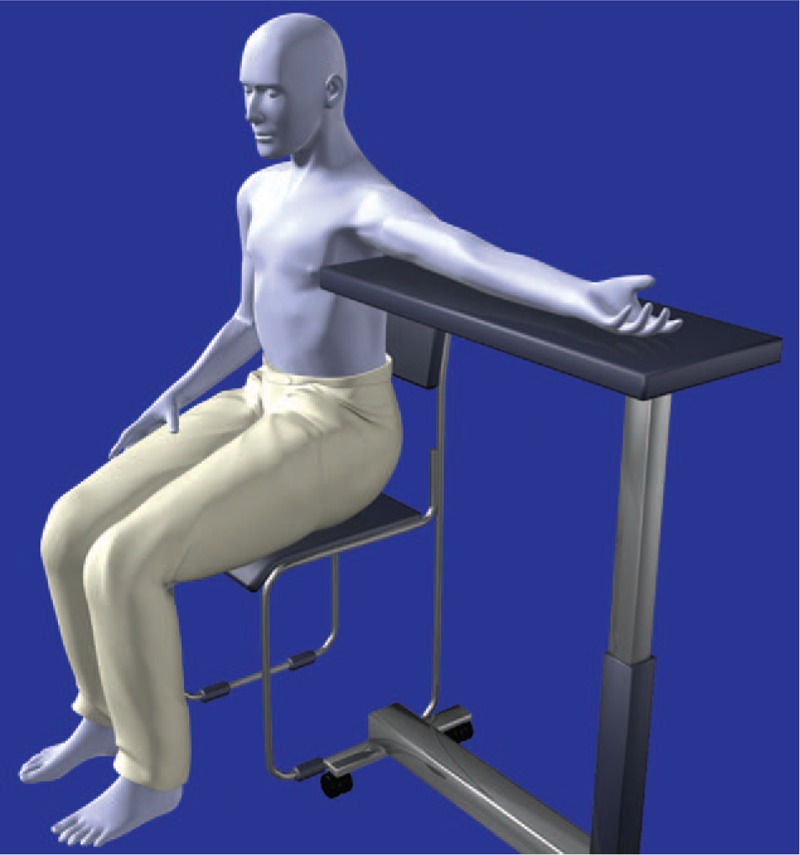
Schematic of the patient in the sitting position during placement of a peripherally inserted central catheter. Successful placement of a peripherally inserted central catheter required adjustment of the height of the table to allow the patient's arm to abduct nearly 90°, as well as to allow the medial side of the patient's arm to face upwards.

The right subclavian vein was suspected to exhibit narrowing, based on computed tomography scan images (Fig. 1). Thus, the anesthesiologists chose the left arm for insertion of a PICC, and found that the medial brachial vein exhibited sufficient size (diameter 4 mm) to place a PICC with ultrasound examination. The anesthesiologists allowed the patient to assume the sitting position, and placed the patient's arm on an over-bed table. PICC placement (4.5 Fr, double lumen, Argyle PICC Kit, Nippon Covidien, Inc., Tokyo, Japan) was performed using ultrasound guidance (6-15 MHz, SonoSite Edge, SonoSite Japan Co., Tokyo, Japan) with maximal sterile barrier precaution (Fig. 2). Cannulation was performed without complications. The technique is summarized as follows (Fig. 3).

(1) The patient was allowed to sit on chair with a backrest.
(2) The patient's stability was ensured (e.g., with aid from an assistant or nurse).
(3) The patient's arm was raised and cleaned with disinfectants (1% chlorhexidine alcohol).
(4) The adjustable over-bed table was covered with a disinfected drape.
(5) The patient's arm was placed on the table.
(6) The arm was allowed to abduct approximately 90° via adjustment of the height of the table.
(7) The operator wore a cap, mask, and surgical gown (maximal sterile barrier precaution).
(8) The assistant placed a towel between the drape and table to ensure that the medial side of the patient's arm faced upwards.
(9) The ultrasound probe was covered with a sterile plastic cover.
(10) Local anesthetic (1% lidocaine 3 mL) was injected with a 25-G needle.
(11) A 20-G over-the-needle catheter (48 mm in length) was inserted into the skin.
(12) The vein was accessed using a short-axis out-of-plane approach; the anterior wall of the vein was penetrated using a long-axis in-plane approach.
(13) The PICC was placed using the modified Seldinger technique.

**Figure 4:**
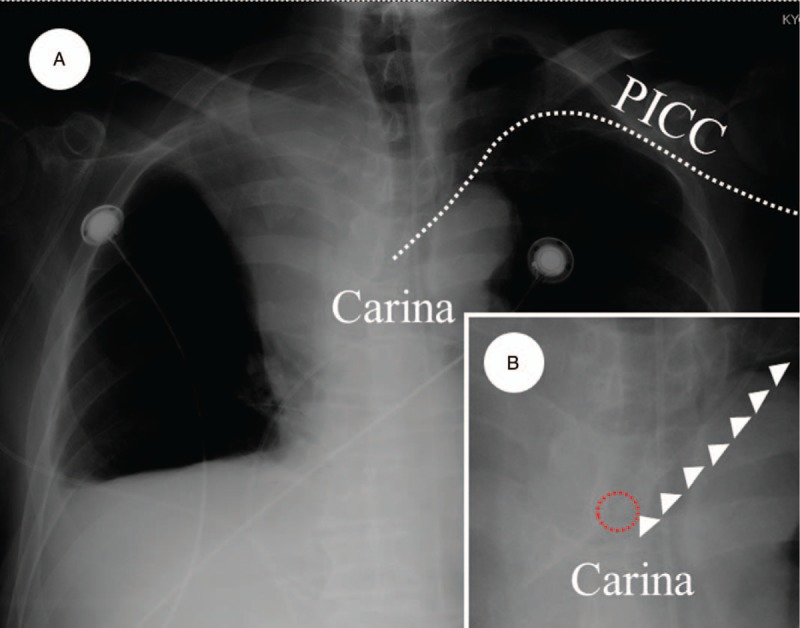
Chest roentgenography confirming the position of the catheter tip. (Panel A) The catheter tip of the peripherally inserted central catheter is located in the left brachial vein. (Panel B) Magnified chest roentgenography shows that the catheter tip of the peripherally inserted central catheter is present in the left brachial vein. White arrows indicate the peripherally inserted central catheter. Red interrupted circle shows the catheter tip. PICC = peripherally inserted central catheter.

**Figure 5:**
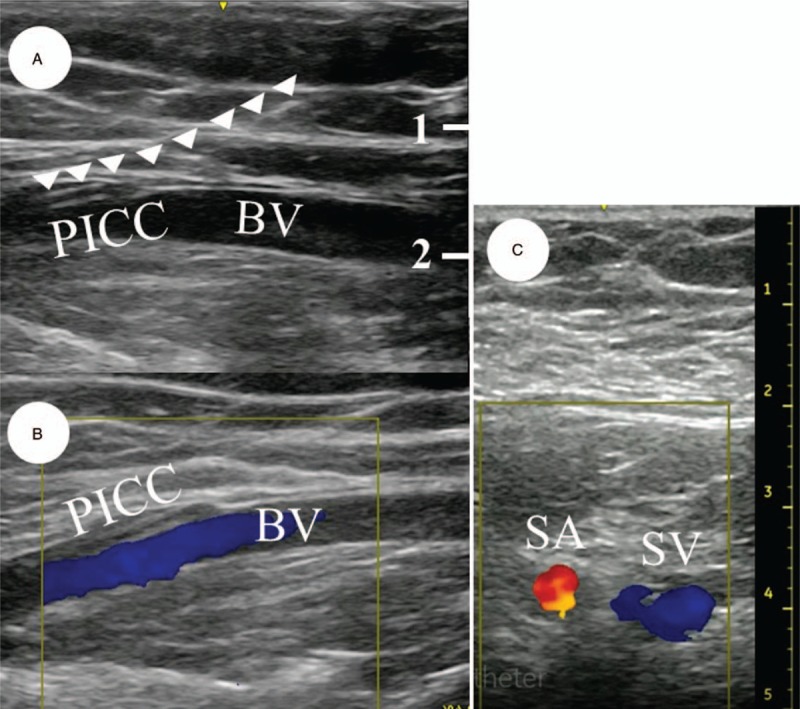
Blood flow of left brachial and left subclavian veins. (Panel A) The peripherally inserted central catheter is present in the left brachial vein. White arrows indicate the peripherally inserted central catheter. (Panel B) Blood flow of the brachial vein is visible with color Doppler ultrasonography. (Panel C) Blood flow of the subclavian vein is visible with color Doppler ultrasonography. BV = brachial vein, PICC = peripherally inserted central catheter, SA = subclavian artery, SV = subclavian vein.

The anesthesiologists attempted to locate the catheter tip in the inferior vena cava or upper right atrium; however, the catheter tip could not proceed to the vena cava. Therefore, the catheter tip was placed in the left brachiocephalic vein (Fig. 4). The left brachial vein and left subclavian vein appeared to maintain blood flow after PICC placement (Fig. 5).

Continuous infusion of noradrenaline and opioid therapy was initiated through the PICC. The patient's orthopnea was slightly ameliorated, and he could sleep at night; however, he remained in the sitting position. The patient died 11 days after insertion of the PICC due to respiratory failure. The PICC was used without difficulty until his death.

## 4 Discussion

The concept of PICC insertion in a patient who has assumed the sitting position is simple and feasible; it is unclear why the technique has not been previously reported. However, many clinicians may prefer to use centrally inserted central venous catheters, rather than PICC, in patients with acute heart failure. Recently, PICC has been used for venous access in critically ill patients.^[3]^ Clinicians must consider the risks of catheter-related complications, which comprise deep vein thrombosis and bloodstream infection, when selecting the type of catheter. No differences have been reported in rates of infectious complications between PICC and centrally inserted central venous catheter approaches in critically ill patients.^[4]^ However, there remains disagreement regarding rates of thrombotic complications between these approaches for critically ill patients.^[4,5]^

Placement of a centrally inserted central venous catheter involves a risk of air embolism when patients are in sitting or upright positions.^[6,7]^ Brederlau et al^[8]^ reported successful ultrasound-guided internal jugular vein catheterization with a patient position of 30° dorsal elevation under mechanical ventilation. In that case, positive end-expiratory pressure (at least 10 cmH_2_O) may have been effective in prevention of air embolism. Tokumine et al^[9]^ reported successful ultrasound-guided internal jugular vein catheterization in a patient with congestive heart failure and acute renal failure in a 45° head-up tilt position. In that case, congestive heart failure caused increased pressure in the internal jugular vein, despite use of the 45° head-up tilt position. There was no report of PICC-related air embolism.^[10]^ The insertion site for a PICC is a peripheral vein, in which pressure is typically higher than atmospheric pressure. This may explain why no air embolism has occurred during PICC insertion. Thus, a PICC may be safely inserted in a patient who has assumed the sitting position.

In summary, we placed a PICC in a patient who had assumed the sitting position, in order to treat congestive heart failure. The technique of inserting a PICC in a patient who has assumed the sitting position is simple and feasible. This approach may be useful for patients in whom central venous access is needed, but the supine position cannot be achieved.

## Author contributions

**Conceptualization:** Joho Tokumine.

**Data curation:** Rena Matsuda.

**Investigation:** Rena Matsuda.

**Methodology:** Joho Tokumine.

**Project administration:** Takayuki Asao.

**Supervision:** Tomoko Yorozu, Takayuki Asao.

**Validation:** Tomoko Yorozu.

**Writing – original draft:** Shingo Mitsuda.

**Writing – review & editing:** Joho Tokumine.

Joho Tokumine orcid: 0000-0003-3481-2085.
